# Redrawing the Map of Great Britain from a Network of Human Interactions

**DOI:** 10.1371/journal.pone.0014248

**Published:** 2010-12-08

**Authors:** Carlo Ratti, Stanislav Sobolevsky, Francesco Calabrese, Clio Andris, Jonathan Reades, Mauro Martino, Rob Claxton, Steven H. Strogatz

**Affiliations:** 1 Senseable City Lab, Massachusetts Institute of Technology, Cambridge, Massachusetts, United States of America; 2 Centre for Advanced Spatial Analysis, University College London, London, United Kingdom; 3 BT Group, Ipswich, United Kingdom; 4 Department of Mathematics, Cornell University, Ithaca, New York, United States of America; Indiana University, United States of America

## Abstract

Do regional boundaries defined by governments respect the more natural ways that people interact across space? This paper proposes a novel, fine-grained approach to regional delineation, based on analyzing networks of billions of individual human transactions. Given a geographical area and some measure of the strength of links between its inhabitants, we show how to partition the area into smaller, non-overlapping regions while minimizing the disruption to each person's links. We tested our method on the largest non-Internet human network, inferred from a large telecommunications database in Great Britain. Our partitioning algorithm yields geographically cohesive regions that correspond remarkably well with administrative regions, while unveiling unexpected spatial structures that had previously only been hypothesized in the literature. We also quantify the effects of partitioning, showing for instance that the effects of a possible secession of Wales from Great Britain would be twice as disruptive for the human network than that of Scotland.

## Introduction

Do regional boundaries defined by governments respect the more natural ways that people interact across space? Beyond its fundamental importance in economic geography [1]–[4], this question underlies many conflicts and struggles for regional independence across the world, such as those that have been recorded across parts of Great Britain over the past decades. To estimate the strength of inter- and intra-regional transactions, traditional analyses have relied on aggregate parameters such as local labour market data, commuter or travel flows and other indexes of accessibility and socioeconomic status [5]–[9]. Here we propose a new, more fine-grained approach to regional delineation, based on analyzing networks of billions of individual human transactions that have recently become available [10]. Given a geographical area and some measure of the strength of links between its inhabitants, we show how to partition the area into smaller, non-overlapping regions while minimizing the disruption to each person's links. We tested our method on the largest non-Internet human network, composed of 20.8 million nodes inferred from a large telecommunications database in Great Britain [11], [12]. Our partitioning algorithm yields geographically cohesive regions that correspond remarkably well with traditional maps and with existing commuting and administrative data. The most striking differences are that Wales and parts of Yorkshire become merged into regions dominated by the major cities of the West and East Midlands, respectively. Our approach could be extended to other large-scale data sets arising in economic geography, urban planning and transportation studies, potentially creating a new type of regional analysis that more closely reflects patterns of human interaction.

We started with a telephone data set containing 12 billion calls over a one-month period, estimating more than 95% coverage of the Great Britain's residential and business landlines in that quarter. Using these data and the methodology explained in Text S1, we inferred a network of roughly 20.8×10^6^ nodes and 85.8×10^6^ undirected links. To safeguard personal privacy, individual phone numbers were anonymized by the operator before leaving storage facilities. Also, each caller's geographic location was specified at the level of spatial units based on a geographic agglomeration of sub-regional switching facility groups (covering 49 km^2^ on average). Thus the geographic agglomeration acts as a kind of mask, preventing us from being able to pinpoint a customer's address, neighbourhood or village.

**Figure 1:**
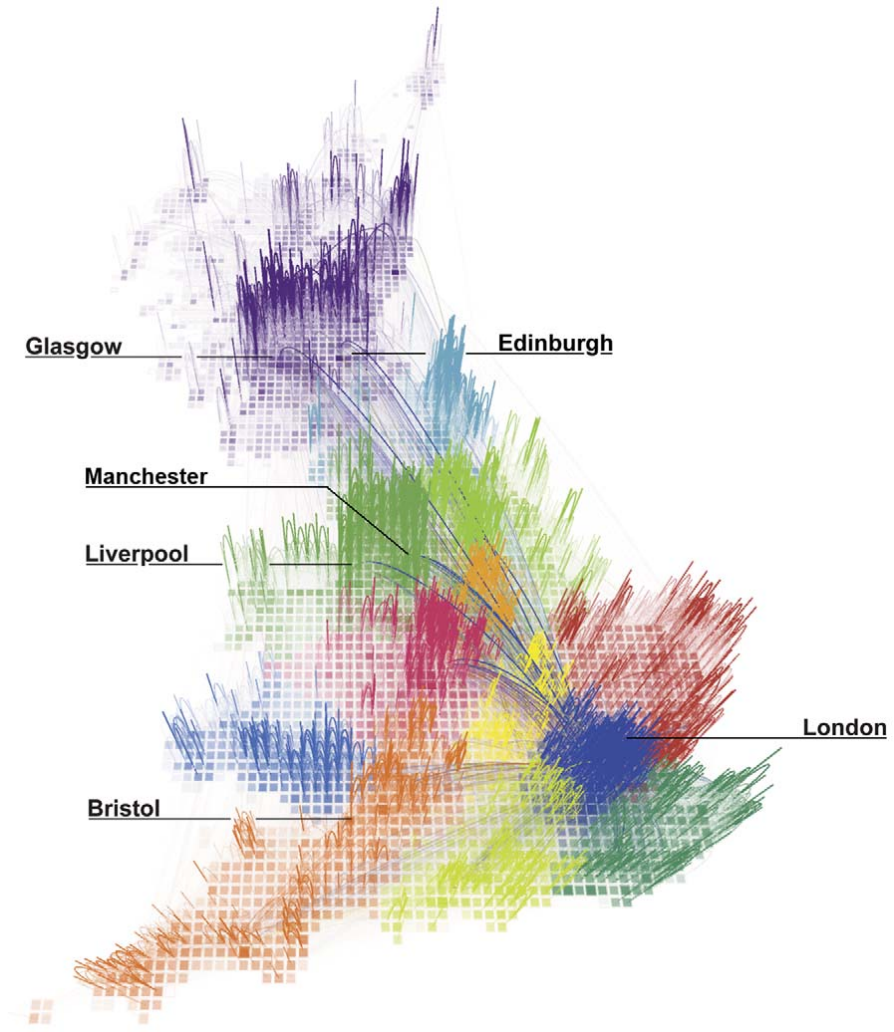
The geography of talk in Great Britain. This figure shows the strongest 80% of links, as measured by total talk time, between areas within Britain. The opacity of each link is proportional to the total call time between two areas and the different colours represent regions identified using network modularity optimisation analysis.

We assumed that the above network is a measure of human interactions at an individual level over all of Great Britain (see discussion in Text S1 and below) and aggregated it into a grid of 3,042 square pixels, each with dimensions 9.5 km by 9.5 km. We treated each pixel as a spatial node and measured its connection strength to every other pixel, thereby deriving a matrix of the total bidirectional traffic between each pair of spatial nodes in the geographic network (Fig. 1). The resulting network of telephone traffic gives an indication of how tightly the thousands of different parts of Great Britain are connected, pixel by pixel. Please note that connection strength was calculated using total call time, hence taking into account the local population density.

## Results and Discussion

The question naturally arises: What is the best way to group these pixels into larger regions? A similar question has been a focus of network research over the past decade; there one seeks the best way to partition a network into separate, non-overlapping communities [13]–[18]. The leading approach is based on optimizing the network's “modularity” [15]. High modularity values occur when the network is subdivided such that there are many links within communities and few between them, as compared to a randomly generated network with otherwise similar characteristics.

However, we are not trying to partition the network itself, but rather to use the network's characteristics to partition the geographic space underneath the network's topology while guaranteeing spatial adjacency, one of the essential features of a geographic region.

Nonetheless, we felt it might be instructive to ignore the adjacency constraint initially, to see what sorts of regions would be obtained. Following Newman's approach as a baseline, we applied his spectral optimization algorithm [16]. Note that it was important to include loop edges (as proposed in [19]) in our analysis as it allowed us to correctly represent the human network from which we started (see Text S2).

**Figure 2:**
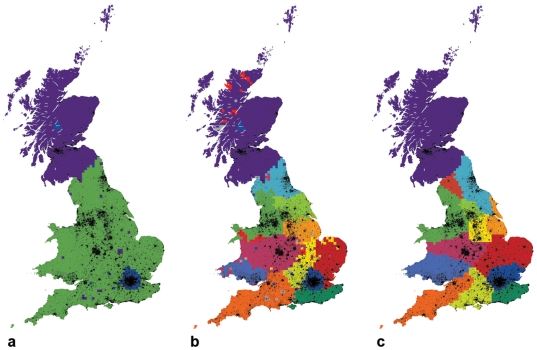
Defining regions through the spectral modularity optimization of telecommunications networks. a - even with just three regions we obtain a total modularity of 0.31, indicating a fairly good network partitioning. b - the final partitioning of Great Britain yields a modularity of 0.58. c - further fine tuning according to the process suggested by Newman [16] increases the modularity to 0.60.

After two iterations of the algorithm, a surprisingly accurate map of the Greater London region emerged, along with an area corresponding to Scotland, with just a few detached pixels scattered across the rest of Great Britain (Fig. 2 (a) and (b)).

With subsequent iterations the modularity increased, ultimately converging to a maximum of 0.58, indicative of a good partitioning compared to the randomized network, as mentioned in [15], [20]. The resulting subdivision had 23 communities, 13 of which were clearly delineated geographically, although some scattered pixels and fuzzy boundaries remained. To determine if these artefacts were due to noise produced by the heuristics of spectral partitioning, we next fine-tuned the spectral partitioning algorithm in a manner suggested by Newman [16], iteratively moving pixels from one region to another to maximize overall modularity (see Text S3). When applied to our data, this process removed the fuzzy boundaries, attached the scattered pixels to their nearest neighbours, and increased the modularity to 0.60.

Figure 2(c) shows the resulting map. Its regional cohesiveness is unexpected: we began by looking at the human network as a topological entity with no geographical constraints, but uncovered clear regions in space that respect spatial adjacency. Apparently the telecommunication links between individuals—and the interpersonal transactions that they capture—are so intertwined with geographical space that partitioning at a network-topological level produces a very accurate partitioning of geographic space. Compared to previously suggested distance-decay models of telecommunication in space [21]–[24], our technique for partitioning shows that not only population distribution in space but also regional boundaries affect the patterns of communication. They also seem to confirm the spatial cohesiveness of partitions defined on mobility networks at an aggregate level, such as airplane connections and banknote movement [25], [26].

**Figure 3:**
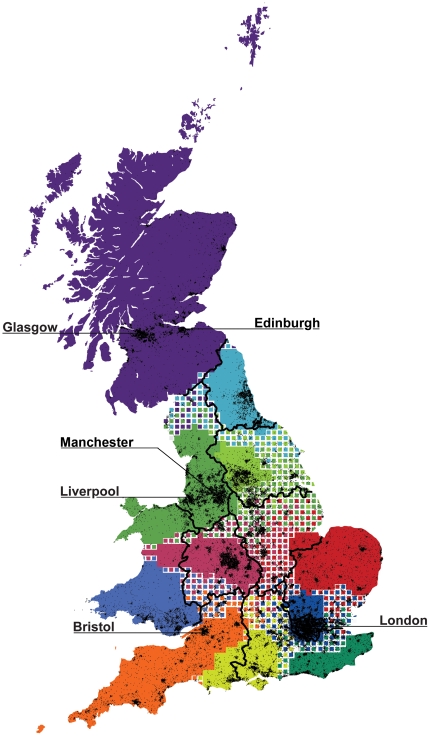
The core regions of Britain. By combining the output from several modularity optimization methods we obtain the results shown in this figure. The thick black boundary lines show the official Government Office Regions partitioning together with Scotland and Wales. The black background spots show Britain's towns and cities, some of which are highlighted with a label.

Before embarking on the detailed examination of our regions, however, we should check how stable our boundaries are. As it has been shown [13], [27], a modularity function such as ours is likely to have exponentially many local maxima, and these maxima typically have different clustered structures. Our partition is likely not to be the global maximum and there are probably alternative local maxima with a high modularity score. What would the corresponding boundaries be? To find out we implemented several modularity partitioning methods (see especially Figure S1 and Text S3). The results are reassuring: there is indeed some variation along the boundaries, but we always find cohesive regions centred approximately in the same place. Also, if we intersect all regions obtained with the different methods, we find 11 stable “cores” that are always separated from each other by “peripheral” regions that lie at the boundaries and have somewhat ambiguous associations (Fig. 3). It should be noted that these “cores” highlight very densely populated areas and contain the great majority of Great Britain's population (85%). Conversely the peripheral regions are very sparsely inhabited. The regional partitioning is also robust with respect to uncertainty in the data, as proven by subsampling (see Text S4), and seems indicative of a highly modular network [20], [27], as seen by comparison with many null models that have an average modularity score of less than 0.02 (see Text S5). We recognize the limits of resolution due to the modularity definition [28]. As we are interested in detecting large regions comparable to the official administrative ones, our analysis did not suffer of this issue. However, multi-resolutions methods could be used to detect smaller robust communities (see [17]).

Another interesting point is that the core map based on human interactions divides Great Britain into approximately the number of “official” Nomenclature of Territorial Units for Statistics 1 (NUTS) British regions (11) —with boundaries that approximately coincide with the traditional ones (Fig. 3). Many of the telecom regions—those corresponding to Scotland, South West, London and the East of England—closely match the forms of historically and administratively important regions. In fact, on average about 80% of pixels fall within a corresponding (by largest overlap) telecom region. While not surprising, this finding seems to corroborate our method: we would indeed expect an agreement between the administrative boundaries and those found from human interaction, as they probably evolved together, over many centuries of mutual interplay—cohesive patterns within society promoting change in administrative boundaries and the latter, in turn, affecting human interaction.

The most obvious difference between the two maps is that Wales, and to a lesser extent Yorkshire, seem to have been incorporated into regions dominated by the major cities of the West and East Midlands regions, respectively. Moreover, we have also “found” a new region developing to the west of London. The first finding supports hypotheses that have long circulated in the transport and regional studies literature: detailed commuting data from the 2001 census was used to generate regions where 95% of trips are internal to that region, finding that Wales, in spite of its unique cultural and linguistic heritage, is well integrated with its English neighbours to the East [29]. Also, the resulting northern and southern Welsh regions match extremely well with our maps. The second finding, of a new region just west of London, corroborates an earlier study of a ‘Western Crescent’ of high-tech activity [30]: a cohesive area that generally scores extremely well in measures of economic activity and low levels of deprivation, as measured by Gross Value Added (GVA) and qualifications (NVQ) for Berkshire, Buckinghamshire, and Oxfordshire [31]. Our partitioning, in short, seems to capture human interaction more accurately than the official NUTS regions. We also overlaid a map of modern English-only dialects [32]. Even if the boundaries of 16 dialects were not well defined, we could informally estimate that the East of England and, in particular, East Anglia matched up fairly well with our corresponding region (around 60% overlap between regions), although the overall overlap between telecom regions and corresponding - by largest overlap - dialects regions was only around 25%. This is what we would expect in country that has undergone centuries of linguistic integration.

There are other metrics for which the partitioning scores better than NUTS. Per our initial hypothesis our regions would produce fewer disturbances to the network of human interaction. This can be seen in Text S3, where we show that boundaries obtained with all modularity partitioning methods always cut fewer ties across the network. Another measure by which our partitioning scores better is that our predicted boundaries cross areas with very low population density (50% that of the official boundaries).

The above partitioning of Great Britain using telecommunication data also suggests the extent to which each region is integrated into the country as a whole. To measure this, we calculate the call time ratio, defined as the percentage of time a region talks to itself. By this measure, Scotland is the region least connected to the rest of Great Britain, followed by North Wales, South Wales and Greater London. What is particularly striking about Scotland is that the call time ratio is 76.7%, meaning that just 23.3% of all call time placed or received in Scotland goes to or comes from another part of the country (as a comparison in a random network we would have only 37% call time ratio). Scotland appears to be loosely coupled with the rest of Great Britain in a way that Wales emphatically is not. In other terms, if Scotland and Wales were to become independent from the UK, and if the detrimental effect of the secession were considered proportional to the number of external connections, the effect on people would be approximately twice more disruptive on Wales than Scotland.

All of the above analysis is based on the pattern of landline calls, but our method could easily be used on other networks in the future: data from mobile phones could be an indicator of more personal (as opposed to household and business-oriented) human interaction [33], while databases from credit card companies could highlight commercial links between individuals. One could even imagine applying a similar analysis to the movement patterns of each individual, and determine boundaries that would minimize their disturbance [34]–[36]. All together, these approaches could lead to a new perspective in regional studies, transportation planning and economic geography.

## Supporting Information

Figure S1 Defining regions through the spectral modularity optimization. Results of five different modularity optimization algorithms.
(5.66 MB TIF)

Text S1 Inferring the network of human interactions from calling data.
(0.05 MB DOC)

Text S2 Definition of modularity.
(0.19 MB DOC)

Text S3 Comparing different modularity optimization methods.
(0.06 MB DOC)

Text S4 Subsampling the network data.
(0.04 MB DOC)

Text S5 Comparison with null model.
(0.04 MB DOC)
