# Supplementary figures and images for: Redrawing the Map of Great Britain from a Network of Human Interactions
Source: PLoS One. 2010 Dec 8;5(12):e14248. doi: 10.1371/journal.pone.0014248 (PMC2999538; doi:10.1371/journal.pone.0014248)

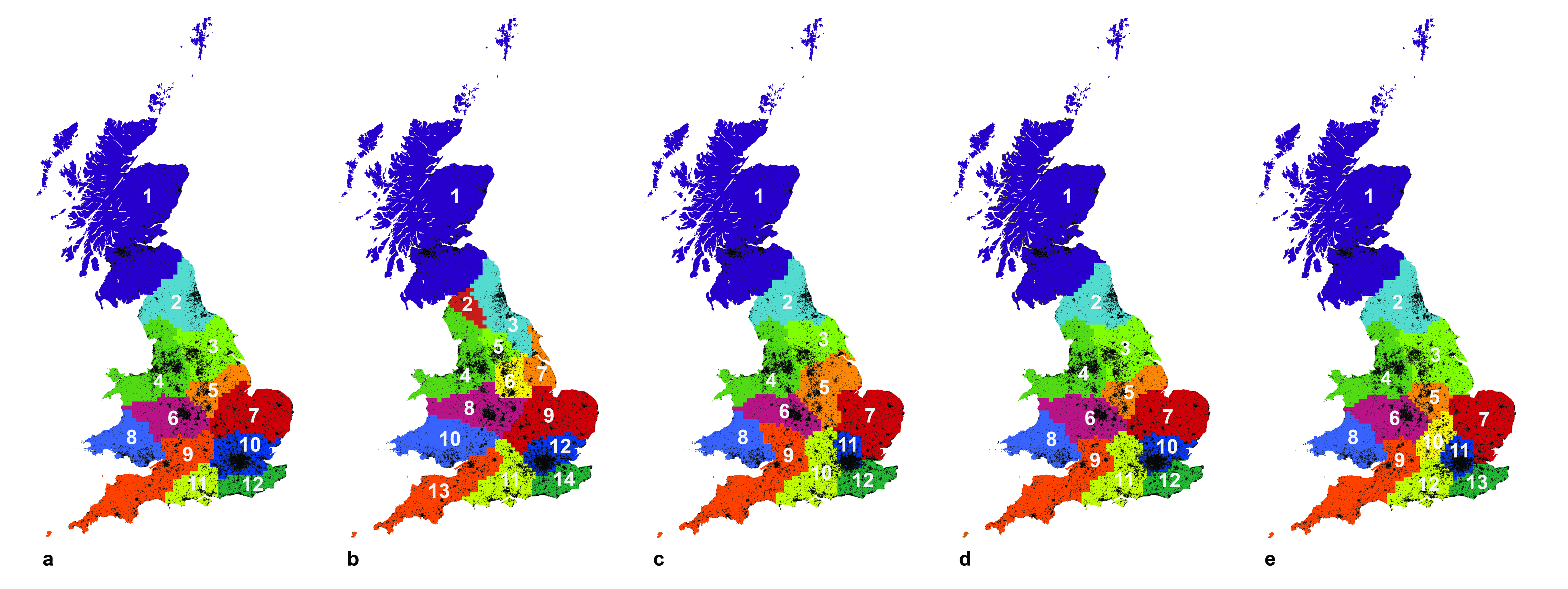

Supplement: Figure S1 — Defining regions through the spectral modularity optimization. Results of five different modularity optimization algorithms. (5.66 MB TIF) [file pone.0014248.s001.tif]
