# Supplementary material for: Redrawing the Map of Great Britain from a Network of Human Interactions
Source: PLoS One. 2010 Dec 8;5(12):e14248. doi: 10.1371/journal.pone.0014248 (PMC2999538; doi:10.1371/journal.pone.0014248)
Supplement: Text S1 — Inferring the network of human interactions from calling data. (0.05 MB DOC) [file pone.0014248.s002.doc]

**Inferring the network of human interactions from calling data**

We used a data set of landline telephone calls containing 12 billion records of individual calls over a one-month period[[1]](#footnote-2). To safeguard personal privacy, individual phone numbers were anonymized by the operator before leaving storage facilities. Also, each caller’s geographic location was specified at the level of spatial units based on a geographic agglomeration of sub-regional switching facility groups (covering 49 km^2^ on average). Thus the geographic agglomeration acts as a kind of mask, preventing us from being able to pinpoint a customer’s address, neighbourhood or village.

We considered only phone numbers that could be located in Great Britain, filtering out callers from other networks (including wireless) as well as calls to and from overseas. To avoid bias due to call centers, we also filtered out one-way callers, keeping reciprocal calls. In other work where people have focused on extracting a ‘social network’ it has been important to eliminate nodes with a high degree as well, indicative for instance of businesses. In our case, however, our goal is not to create a social network, but to capture all types of interactions, so we did not eliminate nodes with a high degree.

From these reciprocal calls, we inferred a network of 20.8 million nodes and 85.8 million undirected links. The analysis of this data partly avoids common bias since:

- It relies on an almost complete view of the communication network - The data contains more than 95% of the residential and business landlines in the country[[2]](#footnote-3).
- The number of nodes is uniformly representative of the population across cities and towns.
- Caller locations are not specified by users as in other work [1], but based on the actual endpoints of landlines.

Given these facts, we may conclude that our network is a fairly unbiased proxy of landline-based human interaction in Great Britain.

The average and median numbers of neighbors per node are 9.57 and 5 respectively, although a few nodes (hubs) have a very large number of neighbors. The network shows well-known “small world” properties [2]. By sampling 10,000 nodes from the largest connected component at random and computing the shortest path distance for each of them, we evaluated an average distance of 6.21, in line with the literature [3].

One could ask if the lack of mobile phone data might bias the results. If mobile phone users were more highly local in their behavior than landline users (or vice versa), our results might well be altered. We do not have mobile phone data for Great Britain to test this issue. However, we have compared the characteristics of our network with a similar one generated for another European country, and found very similar behavior in calling patterns over distance [4].

For the purpose of this research, we aggregated the created network into a grid of 3,042 square pixels, each with dimensions 9.5 km by 9.5 km. We treated each pixel as a spatial node and measured its connection strength to every other pixel as the sum of the length of all calls between phones located in the respective pixels. The resulting network of telephone traffic gives an indication of how tightly the thousands of different parts of Great Britain are connected, pixel by pixel. Please note that connection strength was calculated using total call time, hence taking into account the local population density. Other measurements could be used instead of the total call time, such as the total number of calls or the total number of reciprocal pairs. We selected as measurement the total call time as it measures the overall exchanged information. We note however, that the other two mentioned measures are linearly related to the selected one, with r^2^ of 0.60 and 0.75 respectively.

We could also consider other definitions of strengths such as normalizing the total call duration by the number of phone numbers in each of the pixels. This would lead to a network representing the expected connection between two randomly selected people living in different areas of Great Britain, and so to a partitioning problem formulation looking at individual human interaction compared to overall exchanged information.

**References**

1. Lambiotte, R. et al. (2008) Geographical dispersal of mobile phone communication networks, Physica A, 387, 5317.
2. Watts, D.J., Strogatz, S.H. (1998) Collective dynamics of ‘small-world’ networks, Nature 393, 440-442.
3. Milgram, S. (1967) The small world phenomenon, Psychology Today 1, 60-67.
4. Krings, G., Calabrese, F., Ratti, C., Blondel, V.D. (2009) Urban gravity: a model for inter-city telecommunication flows. *J. Stat. Mech.: Theory and Experiment* 07, 1-8.

1. The anonymized call logs were recorded by the network operator as required by law and for billing purposes and not for the purpose of this project. Although these communication data and the exact location of the telephone exchanges are not publicly available, the aggregated network of human interaction used in this paper is available from the first author. [↑](#footnote-ref-2)
2. The percentage corresponds to the network operator’s estimated number of landlines involved in calls during the study period. [↑](#footnote-ref-3)
