# Supplementary material for: Redrawing the Map of Great Britain from a Network of Human Interactions
Source: PLoS One. 2010 Dec 8;5(12):e14248. doi: 10.1371/journal.pone.0014248 (PMC2999538; doi:10.1371/journal.pone.0014248)
Supplement: Text S2 — Definition of modularity. (0.19 MB DOC) [file pone.0014248.s003.doc]

**Definition of modularity**

Consider a weighted, symmetric network of
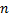
 nodes, with given weights of edges between node
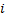
 to node
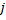
 denoted by
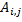
 (with
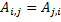
). Suppose that nodes also possess loop edges to themselves, i.e., we assume that
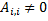
 is possible. We refer to the symmetric matrix A of the values
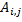
 as a weighted adjacency matrix of the network. Introduce, for each node
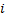
 its strength as the total weight of all the edges connected with this node:
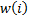
=
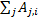
. Also define the total network adjacency matrix weight
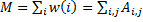
 (here and further
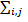
denotes a sum by all pairs of
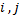
 where pairs
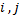
 and
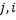
 for
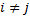
 are considered separately while the pair
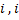
 is considered only once).

Consider a suggested partitioning for which
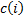
 denotes the index of the community to which node
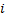
 belongs. Then we perform our calculations using a modularity function defined as


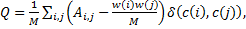


where the
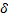
 is the Kronecker symbol, equal to 1 if
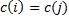
 and 0 otherwise.
