# Supplementary material for: Redrawing the Map of Great Britain from a Network of Human Interactions
Source: PLoS One. 2010 Dec 8;5(12):e14248. doi: 10.1371/journal.pone.0014248 (PMC2999538; doi:10.1371/journal.pone.0014248)
Supplement: Text S3 — Comparing different modularity optimization methods. (0.06 MB DOC) [file pone.0014248.s004.doc]

**Comparing different modularity optimization methods**

A variety of methods have been proposed for modularity optimization (see review articles in [1] and [2]). We have compared the regions resulting from a subset of five different modularity optimization methods, and studied the differences among them:

1. CNM fast greedy optimization algorithm [3].
2. Spectral algorithm with Kernighan–Lin like refinement [4].
3. Improved spectral algorithm with Kernighan–Lin like refinement (see the original one in [4]), in which we allow a new operation: a shift from one community to another (not necessarily empty) community.
4. Combined optimization algorithm, involving three basic operations: (i) splitting a community into two by a spectral technique; (ii) joining two communities (not necessary geographically close); and (iii) shifting a part of one community to another.
5. Best mix. We intersect the regions resulting from all previous methods, and apply the CNM agglomerative method to these subregions to find a higher-modularity partition.

All these algorithms generate cohesive regions (see Figure S1) and modularity values between 0.60 and 0.61.

| **Parameter/Method** | **NUTS1** | **a** | **b** | **c** | **d** | **e** |
| --- | --- | --- | --- | --- | --- | --- |
| **Modularity** | 0.595096 | 0.606853 | 0.606473 | 0.611474 | 0.611636 | 0.613114 |
| **Links cut** | 30.2% | 27.2% | 28.7% | 28.9% | 28.1% | 28.9% |
| **Match with NUTS1** | 100% | 80.0% | 75.3% | 83.7% | 82.3% | 80.3% |
| **Average population density on boundary (people/km^2)** | 411 | 183 | 219 | 243 | 209 | 227 |
| **Regions** | 11 | 12 | 14 | 12 | 12 | 13 |

The table above shows some comparison indexes. The regions all perform better than the official NUTS in terms of modularity, links cut (percentage of call time that traverses different regions) and average population density on the boundary. The number of regions ranges from 12 to 14. However, the different regions have very similar shapes, as can be seen in Figure 2 of the main text, showing the intersection of similar regions in the three partitions. We define these regions as “core” since they are always separated and cover 73% of Great Britain’s area, and 85% of the population. The remaining areas lie at the boundaries and have somewhat ambiguous associations, which depend on the type of algorithm used.

Starting from the regions defined using method **e**, which gave the highest modularity score, we computed the call time ratio for each region, i.e. the percentage of time a region talks to itself. Results are reported in the table below.

| **Region n.** | **Call time ratio** |
| --- | --- |
| 1 | 76,74% |
| 4 | 64,96% |
| 8 | 61,71% |
| 2 | 60,36% |
| 3 | 56,44% |
| 11 | 53,97% |
| 6 | 53,26% |
| 7 | 51,47% |
| 9 | 51,40% |
| 13 | 46,19% |
| 5 | 43,19% |
| 12 | 42,45% |
| 10 | 34,72% |

**References**

1. Fortunato, S. Community detection in graphs. *Phys. Rep.* 486, 75–174 (2010).
2. Porter M. A., Onnela J.-P., and Mucha P. J.. Communities in Networks. *Not. Amer. Math. Soc.* **56**, 1082–1097, 1164–1166 (2009).
3. Clauset A., Newman M. E. J., Moore C. (2004) Finding community structure in very large networks. Phys. Rev. E 70, 066111.
4. Newman, M. E. J. (2006) Finding community structure in networks using the eigenvectors of matrices. Physical Review E 74, 036104.
