# Supplementary material for: Redrawing the Map of Great Britain from a Network of Human Interactions
Source: PLoS One. 2010 Dec 8;5(12):e14248. doi: 10.1371/journal.pone.0014248 (PMC2999538; doi:10.1371/journal.pone.0014248)
Supplement: Text S4 — Subsampling the network data. (0.04 MB DOC) [file pone.0014248.s005.doc]

**Subsampling the network data**

In order to test the robustness of our boundaries against subsampling the data we performed the following uncertainty analysis. Starting with the full network, we randomly removed individual call volumes from the edges. We tested 100 different networks for different levels of removal, from 1 to 99% of the total call volume and used the fast greedy algorithm [1] to perform the partitioning. We found that all partitions have exactly 12 regions whose boundaries closely match those of the initial network. The modularity value is stable at 0.6068 until the remaining edge weight reaches 10%, where the score slightly increases (to 0.607) due to the disappearance of some nodes (caused by the removal of the associated edges).

**References**

1. Clauset A., Newman M. E. J., Moore C. (2004) Finding community structure in very large networks. Phys. Rev. E 70, 066111.
