# Supplementary material for: Redrawing the Map of Great Britain from a Network of Human Interactions
Source: PLoS One. 2010 Dec 8;5(12):e14248. doi: 10.1371/journal.pone.0014248 (PMC2999538; doi:10.1371/journal.pone.0014248)
Supplement: Text S5 — Comparison with null model. (0.04 MB DOC) [file pone.0014248.s006.doc]

**Comparison with null model**

We computed the modularity value for 100 random networks preserving the same node weights distribution. On all the tested random networks, the sum of all positive elements of the modularity matrix:

$$A_{ij}-\frac{w_{i}w_{j}}{M}$$

does not exceed 0.166, which gives an upper bound to the maximum value of modularity. Using the fast greedy modularity optimization algorithm [1] on the same random networks we get modularity values around 0.02.

These values are lower than the lower bound modularity we have for the real network i.e. considering all nodes as distinct communities (0.191). They are also much lower than the average 0.6 modularity we found using optimization algorithms, showing that our network is strongly modular, and that the regions we delineate are not generated by coincidence.

**References**

1. Clauset A., Newman M. E. J., Moore C. (2004) Finding community structure in very large networks. Phys. Rev. E 70, 066111.
